# Bridging the Gap in Medical Education: A Qualitative Study on the Perspectives of Japanese Medical Students and Patients on Outcomes-Based Education

**DOI:** 10.7759/cureus.95527

**Published:** 2025-10-27

**Authors:** Daisuke Son, Mieko Homma, Joyce Pickering

**Affiliations:** 1 Department of Community-based Family Medicine, Faculty of Medicine, Tottori University, Yonago, JPN; 2 Department of Medical Education Studies, International Research Center for Medical Education, Graduate School of Medicine, The University of Tokyo, Tokyo, JPN; 3 Department of Health Behavior, Saitama Prefectural University, Koshigaya, JPN; 4 Department of Medicine, McGill University, Montreal, CAN

**Keywords:** medical education, outcome-based education, patient-centered approach, qualitative research, stakeholder perspectives

## Abstract

Introduction: Outcomes-based education (OBE) has transformed medical education by focusing on specific, measurable learning results. However, educators typically formulate these outcomes with little regard for the perspectives of key stakeholders, such as students and patients. This study explored the perceptions of Japanese medical students and patients regarding these outcomes.

Methods: We conducted focus group interviews with 14 medical students and 13 patients from the first author's university. Participants reflected on the eight outcomes of the 2010 edition of the Model Core Curriculum for Medical Education in Japan. Qualitative data analysis was conducted using thematic analysis.

Results: Medical students emphasized the importance of practical education, patient interaction early in their training, and education that bridges knowledge and action, questioning the effectiveness of traditional teaching methods related to professionalism and communication skills. The students also expressed dissatisfaction with simulated learning for team care. In contrast, patients stressed the importance of physicians' empathy and communication skills alongside a patient-centered research approach. They also expressed a desire for a range of ways in which physicians respond to patients as individuals.

Conclusion: The study results have significant implications for outcomes-based medical education. Both medical students and patients questioned the efficacy of the traditional curriculum, notably in teaching professionalism, communication skills, and team care. The findings suggest that medical education outcomes for future physicians should integrate practical application, empathy training, and flexibility.

## Introduction

Outcomes-based education (OBE) is a significant development in medical education that defines clear and measurable learning outcomes for students [1-3]. This approach assesses students' knowledge and skills based on their ability to demonstrate specific competencies. OBE specifies clear educational outcomes that determine various aspects of the curriculum, teaching methods, and assessment processes [3]. OBE is effective in improving student learning outcomes, particularly in critical thinking and problem-solving [2]. Studies suggest that several factors influence OBE effectiveness, including assessment strategies, faculty development, and ongoing evaluation and refinement of the curriculum [1,2]. The World Federation for Medical Education (WFME) has developed global standards for undergraduate and graduate medical education, emphasizing an outcomes-based approach [4,5].

In Japan, the principles of OBE were incorporated into medical education through the development of the Model Core Curriculum (MCC), which defined the competencies required of medical graduates. The MCC for Medical Education was developed by Japan's Ministry of Education, Culture, Sports, Science, and Technology (MEXT) as a guideline for establishing medical education programs [6]. The curriculum emphasizes the competencies and skills that students should acquire by the end of their medical education program instead of knowledge acquisition [7,8]. The MCC for Medical Education in Japan specified the basic qualifications required of graduating physicians for the first time in its 2006 edition, which were later modified in 2010. The qualifications include demonstrating responsibility as a physician, an ability to understand patient-centered viewpoints, communication capabilities, an ability to provide care in teams, comprehensive practice competencies, a commitment to local community medical care, interest in medical research, and skills in self-directed learning [9]. The latest 2022 edition of the MCC lists the following 10 qualities that a medical graduate should have: professionalism, generalism, a lifelong learning approach, an appreciation of research, problem-solving skills, an ability to effectively use information technology, clinical skills, communication skills, an ability to collaborate interprofessionally, and an understanding of the role of medicine in society [10].

However, educators largely lead the formulation of medical education outcomes. Existing research on the perceptions of patients and students, as essential stakeholders in educational outcomes, is insufficient. Although many countries have well-defined learning outcomes in medical education, most evaluations of these outcomes have been conducted from the perspectives of educators or institutions rather than those of patients or students. For example, previous research has examined patients' perspectives on professionalism [11] and medical students' views on their learning environment [12-14], but few studies have directly explored how these stakeholders perceive or evaluate the outcome statements themselves.

Therefore, this study aimed to explore how Japanese medical students and patients perceive the eight learning outcomes defined in the 2010 edition of the MCC for Medical Education, specifically professionalism, patient-centeredness, communication, teamwork, comprehensive practice, community care, research orientation, and self-directed learning. By examining stakeholder perspectives on these outcomes, this study seeks to identify gaps between formal educational goals and lived educational experiences. The findings are intended to inform the design of future curricula by highlighting how learning outcomes can be translated into more experiential, empathy-focused, and contextually relevant educational practices.

While recent revisions of Japan's MCC, including the 2016 and 2022 editions, have begun to involve limited student and patient participation in defining competencies, genuine stakeholder inclusion remains at an early stage. Understanding how these key stakeholders perceive learning outcomes developed historically without their input is educationally essential, as it reveals the extent to which defined outcomes align with, or fail to align with, their lived experiences and expectations.

We define the "gap" as the mismatch between educator-formulated outcomes and the perspectives of primary stakeholders: medical students and patients. This study elicited those perspectives (2014) with reference to Japan's 2010 MCC outcomes and proposes procedures for embedding stakeholder input into outcome statements themselves.

## Materials and methods

Participants and data collection

This study is based on the constructivist paradigm that human knowledge is socially constructed, not discovered. Study participants were selected through purposive sampling as follows. For the medical student sample, we invited all medical students in years 1-6 at the first author's university to participate via email. Participants who responded and consented to participate in the study were included. Email invitations were sent to all 620 medical students enrolled in the university in 2014. A total of 14 students (2.3%) volunteered and participated.

For the patient sample, we emailed members of a simulated patient group affiliated with the first author's university. Participants who responded and reported having a chronic disease were included in the study. A total of 35 simulated patients affiliated with the university's simulated patient program were contacted by email, of whom 13 (37%) consented to participate. No explicit exclusion criteria were applied beyond withdrawal of consent. The patient group consisted of simulated patients affiliated with the university who also lived with chronic conditions. They were selected purposively because they had extensive experience interacting with medical students in educational settings and could articulate reflective perspectives on communication, professionalism, and team care, while also drawing on their own lived experiences as patients.

We conducted four focus group interviews with 14 medical students and three with 13 patients between March and April 2014. Four focus groups were held with students (three to four participants each) and three with patients (four to five participants each). Student and patient groups were conducted separately to ensure open discussion. Each focus group lasted 53-68 minutes and was conducted in a private seminar room at the university. Group dynamics were balanced by alternating speaking turns and ensuring equal opportunity for contribution. Questions asked in the focus group interviews included, "What do you think an ideal physician is like?" and "What do you want from medical education?" Focus group questions were phrased in open-ended form, with neutral prompts such as "Can you tell me more about that experience?" Terms such as "patient-centered care" were introduced only after participants had described their own ideas about good medical practice, ensuring that predefined terminology did not shape their responses. The facilitator avoided evaluative feedback and used reflective listening to promote balanced participation.

In the second half of the interview, we showed participants the eight outcomes of the MCC for Medical Education 2010 edition (demonstrating responsibility as a physician, patient-centered viewpoints, communication capabilities, team care, comprehensive practice competencies, local community medical care, interest in medical research, and self-directed learning). We sought opinions regarding these outcomes and medical education from both medical students and simulated patients. The focus group interviews were recorded by digital recording equipment, and verbatim transcripts were made.

The first author conducted focus group interviews for the research participants. The first author, a physician and faculty member at the medical school, was familiar with the student participants through teaching and with the simulated patient participants through their activities in the educational program. This familiarity may have influenced participant responses. To mitigate such influence, participation was voluntary and unrelated to course evaluation, focus group interviews were conducted outside class hours, and reflexive memoing and peer debriefing were used throughout the study to enhance transparency and minimize bias.

Data were collected in 2014, when the MCC 2010 edition was the official guideline; the 2022 edition is cited only to provide contextual reference to subsequent reforms.

Data analysis

Qualitative data analysis was conducted using thematic analysis [15], which consists of the following six steps: familiarization, coding, generating themes, reviewing themes, defining and naming themes, and writing up the results [15,16]. Data analysis was conducted primarily by the first author. All transcripts were imported into NVivo 12 (QSR International, Burlington, MA) for systematic data management and coding. A codebook defining each code and its inclusion/exclusion criteria was developed during initial coding. Two co-authors independently reviewed portions of the transcripts using the codebook, and discrepancies were resolved through discussion until consensus was achieved. When discrepancies in coding or interpretation occurred, they were discussed collaboratively until consensus was reached through iterative meetings. Two co-authors independently reviewed portions of the transcripts and the developing codebook to ensure analytic consistency. Throughout the analytic process, reflexive memoing and peer debriefing were employed to enhance transparency and reduce potential researcher bias. After the qualitative analysis, we conducted iterative data analysis and discussed the validity of interpretations among researchers for triangulation.

All transcripts were anonymized by a research assistant who was not affiliated with teaching. Identifiable information was removed before analysis, and participants were assigned numerical IDs. The first author analyzed de-identified transcripts to protect participant confidentiality.

We monitored thematic saturation throughout data collection and determined that code-level saturation was reached when no new themes emerged in the final student and patient focus group interviews. This sample size was considered adequate given the study's exploratory purpose and the conceptual richness of the data. In presenting quotations, we indicated whether each excerpt reflected a widely shared theme among participants or an individual but illustrative viewpoint.

Ethical considerations

This study was conducted with the approval of the Ethical Review Committee of the University of Tokyo (Approval No. 10431). Study participants were informed beforehand that their participation was voluntary, and all participants provided their written consent.

## Results

The study comprised 14 medical students and 13 patients. Tables 1, 2 present the participants' backgrounds.

**Table 1:** Background of the medical student participants

ID	Gender	Age	Grade of Medical School	Focus Group Interview Duration (Minutes)
1	Female	20s	4	64
2	Female	20s	4	
3	Male	20s	4	
4	Male	20s	4	
5	Female	20s	4	68
6	Male	20s	5	
7	Male	20s	5	
8	Female	20s	4	
9	Male	20s	2	62
10	Female	20s	3	
11	Female	20s	3	
12	Female	20s	2	53
13	Male	30s	6	
14	Male	20s	5	

**Table 2:** Background of the patient participants

ID	Gender	Age	Past Medical History	Focus Group Interview Duration (Minutes)
1	Female	50s	Uterine myoma, ovarian cyst	57
2	Female	50s	Ovarian cancer	
3	Female	50s	Endometriosis, atopic dermatitis	
4	Male	60s	Hypertension, prostatic hypertrophy	
5	Male	60s	Hypertension	
6	Female	60s	Uterine myoma, breast cancer	60
7	Female	60s	Lung cancer	
8	Female	60s	Ulcerative colitis, rectal cancer	
9	Female	60s	Rectal cancer, chronic thyroiditis	
10	Female	70s	Hypertension	55
11	Female	60s	Dyslipidemia	
12	Female	50s	Hypertension	
13	Female	60s	Diabetes, Hypertension	

The thematic analysis generated 13 themes for medical students and nine themes for patients, organized into categories based on the eight outcomes of the MCC 2010 edition, with another separate category added, as shown in Tables 3, 4.

**Table 3:** Results of the thematic analysis of medical student interviews

Categories	Themes
Demonstrating responsibility as a physician (Professionalism)	Difficulty of teaching/learning professionalism didactically
Patient-centered care	Needs for contact with patients from the early stage
Communication skills	Importance of education in empathy
	Flexibility to accept diverse opinions and perspectives
	Questioning whether communication should be taught in a didactic format
Team care	Simulated learning that feels artificial
Comprehensive practice competencies	Needs for comprehensive practice competencies
	The study of generalism should not overshadow learning about rare diseases
Community medical care	Needs for informal learning outside the campus
	Commitment to wider social issues
Interest in clinical medical research	Needs for research learning applicable to clinical practice
	Questioning whether research learning should be mandatory
Self-directed learning	Needs for opportunity to take responsibility for patients

**Table 4:** Results of the thematic analysis of patient interviews

Categories	Themes
Patient-centered care	Needs for a physician who empathizes with the patient’s perspective
	Needs for compassion and care for patients
	Concern about fixating on the physician's biomedical perspective, rather than the implications for the patients in their social context
	Questions about training in empathy
Communication skills	Needs for communication training
	Needs for physicians with humor and rich sense of humanity
	Needs for physicians who are psychologically fulfilled
Interest in clinical medical research	Patient-centered research perspective contrasting with students' clinically oriented view
Others	Tolerance in education for physicians responding to patients as individuals

Medical students' perspectives

The analysis of medical students' narratives of medical education outcomes generated 13 themes. A description of each category follows.

Demonstrating Responsibility as a Physician (Professionalism)

One theme was generated in the area of professionalism: the difficulty of teaching/learning professionalism didactically. Many students felt that professionalism and responsibility for patients should be learned from practical learning and not from lecture-style learning.

"(As for professionalism), we discuss it in tutorials, and I think it’s meaningful to discuss it at least once, but I don’t understand it if it’s taught from a textbook. And I think it’s closely related to one’s personality and character, so I’m not sure if it’s something that can be taught." (Theme: difficulty of teaching/learning professionalism didactically)

Patient-Centered Care

The category of patient-centered care generated the following theme: the need for contact with patients from an early stage. Although early contact with patients contributes to the development of communication skills, students described it primarily as a way to cultivate empathy and patient-centered attitudes, rather than as a skill-training opportunity.

"I think it is better to do such things as listening to patients’ stories and other people’s stories at an early stage, such as in the first or second year, rather than in the fourth or fifth grade."* *(Theme: need for contact with patients from an early stage)

Communication Skills

Three themes were generated in the area of communication skills: the importance of education in empathy, flexibility in accepting diverse opinions and perspectives, and questioning whether communication skills should be taught in a didactic format. Many students found education on communication and empathy useful and saw value in classes that led to receptivity to diverse perspectives. However, they also questioned the idea of communication being taught in a didactic format.

"I had a clear opinion about it, but when I talked about it in the PBL group, there were many people who said completely different things. So, it was great to have the opportunity to listen to them and realize that my way of thinking was biased." (Theme: flexibility in accepting diverse opinions and perspectives)

"I don’t think it’s necessary to develop communication skills or team-based care in university classes. However, I think that all of these outcomes are necessary before becoming a doctor in terms of realizing their importance." (Theme: questioning whether communication should be taught in didactic format)

Team Care

The team care category generated the following theme: simulated learning that feels artificial. Some students did not appreciate the current method of learning about team medicine and compared the learning simulation about team medicine to a type of game.

"For example, team medicine is not something that can be taught in the classroom. I mean, how are we going to achieve it? But in fact, even if you do this for 100 people in medical school, it will be nothing more than a game of ‘make-believe,’ so I feel that it is no use to ask too much of school education…." (Theme: simulated learning that feels artificial)

Comprehensive Practice Competencies

The category of comprehensive practice competencies generated two themes: the need for comprehensive practice competencies and the study of generalism should not overshadow learning about rare diseases. Many students expressed that comprehensive practice skills are inherently important but also believed that the study of generalism should not overshadow the importance of learning about rare diseases.

"Rather than just knowing the details of a very rare disease, it’s important to have a willingness to learn about these areas. If you don’t have an attitude of not being satisfied with just being able to diagnose comprehensively, no one will be able to diagnose people with very rare illnesses, and there will be no one left to diagnose them." (Theme: study of generalism should not overshadow learning about rare diseases)

Community Medical Care

Two themes were generated in the area of community medical care: the need for informal learning outside the campus and the commitment to wider social issues. Although categorized under community medical care in accordance with the framework of the MCC, students' comments extended beyond local healthcare contexts to include broader social and systemic issues, such as societal inequities and the role of physicians in contributing to social change.

"When deciding on a future career, I think there are people who say, ‘I want to contribute to society in this way,’ or ‘I want to change this kind of system.’ In order to do so, I think it is difficult for them to feel that way unless they know about the problems that exist in society today and the things that are happening in the world today." (Theme: commitment to wider social issues)

Interest in Clinical Medical Research

The area of interest in clinical medical research generated two themes: the need for learning to conduct research applicable to clinical practice and whether research training should be mandatory. Many students were skeptical about all medical students having to learn to conduct research; instead, they considered that students should learn how to apply research findings to be applied to clinical practice.

"Basic medical research is of course necessary, but I would like to see more research related to clinical medicine. Research is not about animal experiments, test tubes, or cells, but rather, in order to provide the best possible medical care to the patients in front of you, you should certainly have that kind of research-oriented thinking and approach." (Theme: need for research learning applicable to clinical practice)

Self-Directed Learning

One theme emerged in the category of self-directed learning: the need for the opportunity to take responsibility for patients. Many students wanted the opportunity to take responsibility for their patients, thereby meeting the need for self-directed learning. By "taking responsibility," students were referring to supervised clinical training experiences in which they could manage their own patients, believing that such opportunities would enhance motivation and promote self-directed learning.

"I think that if we have the opportunity to take responsibility for our own patients in the hospital wards, we will inevitably realize that we will make mistakes without more knowledge taught in class. Therefore, I think it is important to provide opportunities for students to take responsibility and to develop a sense of self-directed learning." (Theme: need for opportunity to take responsibility for patients)

Patients' perspectives

The interviews with patients about medical education outcomes produced nine themes.

Patient-Centered Care

Three themes emerged from patient-centered care: the need for physicians to empathize with patients' perspectives, the need for compassion and care for patients, concern about fixating on the physician's biomedical perspective rather than on the implications for patients in their social context, and questions about training in empathy. Many patients saw a need for education that would foster caring and compassionate health care providers who considered the patients' perspectives; however, some patients questioned whether these attributes could be taught.

"I think it would be good if there were classes or training to help students acquire such insight and consideration, the ability to immediately catch on to what a patient is looking for even after just a short conversation." (Theme: need for compassion and care for patients)

"Doctors may become accustomed to doing the same thing all the time, but for patients, everything is new. I wonder about the perspective of specialists who focus only on the curing the disease and not on the patient’s life." (Theme: concern about fixating on the physician’s biomedical perspective, rather than implications for the patients in their social context)

"I don’t know if it’s called ‘human ability,’ but I think a good doctor has that kind of thing. But that is not something you were educated for. Well, of course there is something that is not only innate but also refined, but I don’t think that they should try to acquire it through education…" (Theme: question about training in empathy)

Communication Skills

In the area of communication skills, three themes emerged: the need for communication training, the need for physicians to have a sense of humor and of humanity, and the need for physicians to be psychologically fulfilled. Some patients saw a need for physicians to be educated in communication skills and to have a rich sense of humor and humanity. In this context, "a sense of humor and of humanity" referred to physicians' capacity to communicate with warmth, empathy, and emotional flexibility, using gentle humor and human connection to ease patients' anxiety.

"I think almost all students today are already aware of the importance of communication skills. However, I feel that they seem to think that studying comes before communication skills." (Theme: need for communication training)

"I want doctors who understand humor and have a sense of humor. And also, a flexible mind. In short, a doctor who has the human nature to say something funny that makes you laugh…" (Theme: need for physicians to have a sense of humor and humanity)

Interest in Clinical Medical Research

In the category of interest in clinical medical research, the following theme was generated: patient-centered research perspective contrasting with students' clinically oriented view. Many patients wanted students to learn research that always assumed the patient as the endpoint, rather than learning about research for research's sake.

"If they focus too much on research, or rather, on developing new drugs and technologies, and forget the perspective that there are patients at the end of the research, I think they will end up doing research for the sake of publishing papers, or something like that. I would appreciate it if they could find a balance between the two." (Theme: need for a research-oriented mindset with a patient-centered viewpoint)

Others

The other category produced the theme of tolerance in education for physicians responding to patients as individuals. Some patients wanted medical education to be flexible enough to allow for a variety of doctors' approaches, rather than having them respond to patients in a rote manner.

"I get the impression that the students’ language and such are quite standardized. In that sense, I get the impression that the students are responding in a rote manner. I think it would be good if there was more variety in the education of doctors." (Theme: tolerance in education for physicians responding to patients as individuals)

## Discussion

The current study gathered perspectives from 14 medical students and 13 patients on medical educational outcomes. Students emphasized the importance of practical, responsibility-driven education, early and frequent patient exposure, and education that creates a bridge from knowledge to action in order to achieve these objectives. However, they questioned the effectiveness of didactic training in the areas of professionalism and communication. Patients stressed the importance of empathy and communication skills in physicians, alongside a patient-centered research approach. They also expressed a desire to be responded to as individuals, rather than receive what they perceive as rote answers. Our study is one of the first to focus on patients' and medical students' perspectives on medical education outcomes, including the perspectives of stakeholders other than educators, which provides new insights into medical education.

Both medical students and patients felt that teaching professionalism, including empathy and communication skills, through the usual didactic methods is not effective. This finding indicates that participants understood learning outcomes not as isolated goals but as competencies that acquire meaning through the educational processes by which they are achieved. Previous research has identified that teaching professionalism and communication skills in medical education can be challenging. For instance, one study suggests that the optimal methods for teaching and assessing professionalism have not been established and that there is a gap between the ideal of professionalism that students are expected to learn and the actual healthcare environment they encounter in practice, leading to student confusion [17]. Similarly, a systematic review indicated that communication skills training programs that included practice with simulated patients or role-playing had more positive effects than those using lectures or discussions only [18]. Consistent with these previous findings, the current study reinforces the need to evaluate pedagogical approaches in these areas, as both students and patients favored experiential and practice-oriented learning over formal instruction.

Moreover, students tended to link learning outcomes with the learning processes through which those outcomes are achieved. This finding suggests that, for students, outcomes such as professionalism are understood not as static end goals but as competencies developed through experiential learning and practical engagement.

Student dissatisfaction with simulated learning for team care calls for a discussion of improving teamwork training methods within the curriculum. A previous study also suggests that delivering IPE as a large-scale activity limited the amount of meaningful interprofessional interaction, and the "artificial" nature of some of their interprofessional activities could limit its value [19]. This finding may also indicate that when learning experiences lack authenticity, students perceive the associated competency, in this case, team care, as less meaningful or important as a learning outcome. It is therefore important to design teamwork training that provides authentic, contextually grounded experiences.

Although categorized under community medical care based on the framework of the MCC, students' reflections extended beyond local contexts to encompass broader issues of social systems, justice, and civic responsibility. This suggests that students understood "community" not merely as a geographic unit but as part of a wider social fabric.

Patients' viewpoints stress the need for physicians to be caring, compassionate, and patient-centered while emphasizing the flexibility in education that allows doctors to engage with patients as individuals. These findings suggest a need to integrate these aspects into medical education, which are recognized as necessary; however, doing so effectively can be challenging. Previous research has shown that empathy and compassion can decline during medical and nursing education due to environmental and systemic factors such as burnout and the hidden curriculum [20,21]. Enhancing physicians' empathy and compassion may be achieved by revising the curriculum to include experiential learning, offering diverse role models, promoting self-reflection, and encouraging faculty flexibility. Although this study was based on the MCC 2010 edition, the subsequent 2022 revision of the curriculum further emphasizes competencies such as professionalism, interprofessional collaboration, and patient-centered communication. The fact that our 2014 findings anticipated these directions indicates that the perspectives of students and patients identified in this study remain relevant across different curriculum editions. The 2022 MCC is therefore referenced not as study material but as a contextual framework demonstrating the ongoing applicability of our results.

Since this study was conducted in 2014, the MCC has evolved through the 2016 and 2022 revisions. The 2022 edition notably incorporated limited representation from students, patients, and public members in its Delphi panel process [22]. However, the number and influence of these representatives remained small, and stakeholder participation is still largely symbolic rather than structural. Our findings thus remain relevant in 2025, as they illuminate the continuing gap between educators' formal outcome definitions and the lived perspectives of students and patients who are directly affected by those outcomes.

Although this study was based on the MCC 2010 edition, later revisions and related literature (2022-2024) are referenced to contextualize how the educational directions identified in our findings remain relevant today.

Our current study has some limitations. First, the participants were selected from one specific university and its associated simulated patient group. The experiences and perspectives of these individuals might not be representative of those from different geographical locations, institutions, or cultural contexts. Second, the first author, a faculty member and physician at the participating university, conducted the focus group interviews. This could have introduced power dynamics into the focus group discussions, affecting participants' willingness to express critical views or speak freely, particularly the medical students. Third, because the patient participants were simulated patients involved in medical education, their perspectives may differ from those of patients without such experience. Therefore, the findings should be interpreted as reflecting the views of educationally engaged patients rather than the broader patient population. While simulated patients' familiarity with medical education may bias them toward idealized expectations, it also provides valuable insight into patient-centered competencies as conceptualized within medical training contexts. Fourth, as this study relied solely on focus group interviews without complementary methods such as surveys or observations, the depth and diversity of perspectives were limited. Future research should integrate multiple data sources to enhance the robustness of findings.

Our study has significant implications for medical education, particularly OBE. The findings can contribute to designing curricula that meet the needs of both students and patients, thereby enhancing the quality of medical education and practice. While this study highlights dissatisfaction among students and patients with current educational goals, the implications should not be interpreted as a call for curricular reform based solely on stakeholder preferences. Instead, medical educators have a responsibility to engage with these perspectives critically and collaboratively, integrating them with professional standards and educational evidence. Developing learning outcomes that reflect both stakeholder input and pedagogical soundness requires ongoing dialogue among educators, students, and patients.

This study provides novel insight into OBE by demonstrating that both medical students and patients interpret educational outcomes through the lens of learning processes rather than as fixed endpoints. This process-oriented understanding, shared across both stakeholder groups, highlights the need for educational designs that integrate experiential and reflective learning. Moreover, the convergence between these early stakeholder perspectives (2014) and directions later formalized in the 2022 MCC suggests the enduring relevance of these findings to contemporary reforms.

## Conclusions

This study revealed that medical students and patients, two key stakeholder groups in healthcare education, hold distinct yet complementary perspectives on learning outcomes. Students valued practical, responsibility-based learning and early patient exposure, while patients emphasized empathy, individualized care, and human connection. These findings underscore that learning outcomes cannot be separated from the educational processes through which they are achieved.

To address the gap between current outcomes and stakeholder expectations, medical educators should collaboratively reinterpret and refine learning outcomes together with students and patients, ensuring that they reflect both professional standards and human-centered values. In practical terms, this implies promoting experiential, reflective, and dialogical approaches, such as early patient contact, interprofessional teamwork grounded in real contexts, and reflective learning environments, that foster professionalism, empathy, and communication. Future curriculum development should thus move beyond prescriptive goals toward co-created OBE that integrates educator expertise with the lived perspectives of learners and patients.

## Appendices

Interview guide (for PATIENTS)

This is a focus group interview using a semi-structured interview method, and while following the pre-determined questions, questions that come to mind during the interview will also be asked as necessary.

Group information/overview of implementation □ Group ID: □ Number of participants: □ Date and time of implementation: □ Location of implementation:

Question content □ What did you think of the doctor in charge based on your previous experiences of seeing a doctor? (Please tell us why you thought the doctor was good or not good.) □ What qualities do you think are necessary for a doctor? □ In the model case of medical education in Japan, the following eight points are set as educational goals as “basic qualities required of doctors”. What do you think of each of these? 1) Responsibility as a physician 2) Patient-centered viewpoints 3) Communication capabilities 4) Team care 5) Comprehensive practice competencies 6) Local community medical care 7) Interest in medical research 8) Self-directed learning □ What do you think is needed in medical education from now on?

Interview guide (for MEDICAL STUDENTS)

This is a focus group interview using a semi-structured interview method, and while following the pre-determined questions, questions that come to mind during the interview will also be asked as necessary.

Group information/overview of implementation □ Group ID: □ Number of participants: □ Date and time of implementation: □ Location of implementation:

Question content: □ Please tell us about your ideal image of a doctor based on your experiences of hospital training and consultations. □ What qualities do you think are necessary for a doctor? □ The following eight points are considered to be the basic qualities required of doctors in the model case of medical education in Japan, and are set as educational goals. What do you think of each of these? 1) Responsibility as a physician 2) Patient-centered viewpoints 3) Communication capabilities 4) Team care 5) Comprehensive practice competencies 6) Local community medical care 7) Interest in medical research 8) Self-directed learning □ What do you think is needed in medical education from now on?

## References

[1] Asim HM, Vaz A, Ahmed A, Sadiq S (2021). A review on outcome based education and factors that impact student learning outcomes in tertiary education system. Int Educ Stud.

[2] Gruppen LD (2012). Outcome-based medical education: implications, opportunities, and challenges. Korean J Med Educ.

[3] Harden RM (1999). AMEE guide no. 14: outcome-based education: part 1-an introduction to outcome-based education. Med Teach.

[4] Prideaux D (2004). Clarity of outcomes in medical education: do we know if it really makes a difference?. Med Educ.

[5] Christensen L, Karle H, Nystrup J (2007). Process-outcome interrelationship and standard setting in medical education: the need for a comprehensive approach. Med Teach.

[6] Ozeki S, Kasamo S, Inoue H, Matsumoto S (2022). Essential milestones in Japanese medical education and data utilization with practical cases from a regional medical university. Int J Inst Res Manag.

[7] Onishi H, Yoshida I (2004). Rapid change in Japanese medical education. Med Teach.

[8] Saiki T, Imafuku R, Suzuki Y, Ban N (2017). The truth lies somewhere in the middle: swinging between globalization and regionalization of medical education in Japan. Med Teach.

[9] Medical Education Model Core Curriculum Expert Research Committee (2025). Model Core Curriculum for Medical Education in Japan (AY 2010 revision). https://www.mext.go.jp/component/b_menu/shingi/toushin/__icsFiles/afieldfile/2011/06/03/1304433_1.pdf.

[10] (2025). Model Core Curriculum for Medical Education in Japan, AY 2022 Revision. https://www.mext.go.jp/content/20250411-mxt_igaku-000028108_00003-2.pdf.

[11] Haney S, Rowland P, Ginsburg S (2022). Patients' perspectives on medical students' professionalism: blind spots and opportunities. Med Educ.

[12] Behkam S, Tavallaei A, Maghbouli N, Mafinejad MK, Ali JH (2022). Students' perception of educational environment based on Dundee Ready Education Environment Measure and the role of peer mentoring: a cross-sectional study. BMC Med Educ.

[13] Litmanen T, Loyens SM, Sjöblom K, Lonka K (2014). Medical students’ perceptions of their learning environment, well-being and academic self-concept. Creat Educ.

[14] Miles S, Leinster SJ (2007). Medical students' perceptions of their educational environment: expected versus actual perceptions. Med Educ.

[15] Braun V, Clarke V (2012). Thematic analysis. APA Handbook of Research Methods in Psychology.

[16] Kiger ME, Varpio L (2020). Thematic analysis of qualitative data: AMEE guide no. 131. Med Teach.

[17] Goodwin AM, Oliver SW, McInnes I, Millar KF, Collins K, Paton C (2024). Professionalism in medical education: the state of the art. Int J Med Educ.

[18] Berkhof M, van Rijssen HJ, Schellart AJ, Anema JR, van der Beek AJ (2011). Effective training strategies for teaching communication skills to physicians: an overview of systematic reviews. Patient Educ Couns.

[19] Rosenfield D, Oandasan I, Reeves S (2011). Perceptions versus reality: a qualitative study of students' expectations and experiences of interprofessional education. Med Educ.

[20] Hojat M, Vergare MJ, Maxwell K (2009). The devil is in the third year: a longitudinal study of erosion of empathy in medical school. Acad Med.

[21] Ward J, Cody J, Schaal M, Hojat M (2012). The empathy enigma: an empirical study of decline in empathy among undergraduate nursing students. J Prof Nurs.

[22] Nishigori H (2024). Medical education in Japan. Med Teach.

